# Acupuncture Treatment for Emotional Problems in Women with Infertility: A Systematic Review and Meta-Analysis

**DOI:** 10.3390/healthcare11202704

**Published:** 2023-10-10

**Authors:** Su-In Hwang, Young-Jin Yoon, Soo-Hyun Sung, Su-Jin Cho, Jang-Kyung Park

**Affiliations:** 1 Department of Korean Medicine, School of Korean Medicine, Pusan National University, Yangsan 50612, Republic of Korea; hwangsi1216@gmail.com; 2 Department of Korean Medicine Obstetrics and Gynecology, School of Korean Medicine, Pusan National University, Yangsan 50612, Republic of Korea; yyj@pusan.ac.kr; 3 Department of Korean Medicine Obstetrics and Gynecology, Pusan National University Korean Medicine Hospital, Yangsan 50612, Republic of Korea; 4 Department of Policy Development, National Institute of Korean Medicine Development, Seoul 04554, Republic of Korea; koyote10010@nikom.or.kr; 5 Research Institute of Nursing Science, Pusan National University, Yangsan 50612, Republic of Korea; chosujin13@gmail.com

**Keywords:** infertility-related emotional problems, infertility, women with infertility, acupuncture, systematic review, meta-analysis

## Abstract

This systematic review and meta-analysis aimed to evaluate the efficacy and safety of acupuncture in treating emotional problems in women with infertility. We searched for randomized controlled trials using acupuncture treatment for emotional problems in women with infertility using 11 databases from their inception to 30 June 2023. The control intervention included no treatment, sham acupuncture treatment, and conventional treatment. The primary outcome was emotion-related rating scales, and the secondary outcomes were total effectiveness rate, quality of life, clinical pregnancy rate, and adverse events. Twelve randomized controlled trials involving 1930 participants were included. A meta-analysis of these studies indicated that, as compared to the control treatment, acupuncture significantly improved the State–Trait Anxiety Inventory, Self-rating Anxiety Scale, Amsterdam Preoperative Anxiety and Information Scale, and Self-rating Depression Scale scores, which were the primary emotion-related outcomes. Furthermore, the meta-analysis demonstrated that acupuncture treatment had a significant effect on the clinical pregnancy rate, which was the secondary outcome. No adverse events were reported in any of the studies. Our findings demonstrate the potential of acupuncture for treating emotional problems in women with infertility. However, well-designed and high-quality randomized clinical trials are required to confirm the effectiveness and safety of acupuncture treatment. The protocol of the current study was registered in PROSPERO (registration number: CRD42020166119).

## 1. Introduction

The World Health Organization defines infertility as the failure to establish a clinical pregnancy after 12 months of regular unprotected sexual intercourse [1]. Worldwide, 15% of reproductive-aged couples are estimated to be infertile [2]. Causes of infertility include female factors, male factors, and unknown or unexplained factors, and couples may have multiple factors contributing to infertility. Common causes of female infertility include ovulatory dysfunction, fallopian tube obstruction, endometriosis, decreased ovarian function, and uterine factors [3]. Common causes of male infertility include semen abnormalities, hormonal disorders, and genetic abnormalities [4]. The prevalence of infertility has been increasing owing to various factors such as marital status, educational achievement, unfavorable lifestyle, increased reproductive disease, artificial abortions, long-term use of contraception, and increased environmental pollution [5,6].

The diagnosis and treatment of infertility cause various physical, emotional, and psychological problems in women with infertility [7,8] and may adversely affect their quality of life and marital status [9]. Women with infertility often experience anxiety, depression, mental stress, and decreased self-efficacy due to repeated failed pregnancies [10,11]. The prevalence of emotional problems in women with infertility varies from study to study; however, according to a recent study, approximately 25–60% of women with infertility experience emotional problems, particularly anxiety and depression [12]. The negative emotions experienced by women with infertility reduce the effect of assisted reproductive procedures [13] and are the major cause of early cessation of infertility treatment [14], thereby resulting in lower chances of pregnancy success and negatively affecting infertility treatment processes. Moreover, infertility-related experiences and pre-pregnancy emotional problems are associated with an increased risk of postpartum depression and physical symptoms [15,16,17].

Therefore, to alleviate negative emotions and emotional pain in women with infertility and increase the possibility of pregnancy, treatment to improve the emotional symptoms of women with infertility has garnered attention. Recently, various psychological interventions, such as psychoanalytic therapy, integrated psychological therapy, cognitive behavioral therapy, and online counseling, have been performed to improve the negative emotional symptoms of women with infertility [18,19].

Acupuncture is a treatment that is widely used in oriental medicine to improve emotional problems [20,21]. Previous studies have shown that acupuncture improves emotional problems such as anxiety [22], depression [23], and mental stress [24] in unspecified people with mental problems; therefore, it is expected to be effective in improving emotional problems in women with infertility. 

Despite extensive research on the therapeutic effect of combining acupuncture and in vitro fertilization to improve pregnancy and implantation rates [25,26,27], the effect of acupuncture on emotional problems in women with infertility remains unclear. A systematic review of acupuncture treatment for emotional problems in women with infertility has been previously published [28]; however, it only addressed anxiety among women with infertility, without exploring other emotional problems. 

Therefore, we performed a systematic review and meta-analysis to evaluate the effectiveness and safety of acupuncture treatment for managing emotional problems in women with infertility.

## 2. Materials and Methods

### 2.1. Protocol and Registration

The protocol for this review was registered in PROSPERO (https://www.crd.york.ac.uk/PROSPERO, registration number: CRD42020166119) and published in the journal [29]. The methodology was established in accordance with the Preferred Reporting Items for Systematic Reviews and Meta-analysis Protocols (PRISMA-P) guidelines [30] (Supplementary Material Table S1).

### 2.2. Data Sources and Searches

#### 2.2.1. Data Sources

We searched 11 electronic databases, including PubMed, EMBASE, Cochrane Library, KoreaMed, Korean Studies Information Service System (KISS), Korean Traditional Knowledge Portal (KTKP), Oriental Medicine Advanced Searching Integrated System (OASIS), Research Information Sharing Service (KISS), National Digital Science Library (NDSL), China National Knowledge Infrastructure (CNKI), and Wan Fang Database, from their inception to 30 June 2023.

#### 2.2.2. Search Strategy

The search terms were as follows: (“infertility” OR “subfertility” OR “subfertile” OR “oligospermia” OR “azoospermia” OR “obstructive azoospermia” OR “genital disease”) AND (“emotion(s)” OR “emotional” OR “mood(s)” OR “feeling(s)” OR “psychological” OR “personality” OR “anxiety” OR “anxious” OR “anxiousness” OR “depression” OR “depressive” OR “stress” OR “distress” OR “distressing” OR “pain(s)” OR “painful” OR “fear(s)” OR “panic(s)” OR “nervousness” OR “self-efficacy” OR “relaxation” OR “adaptation” OR “mental disorder”) AND (“acupuncture” OR “acupressure” OR “electroacupuncture” OR “auricular acupuncture” OR “scalp acupuncture” OR “hand acupuncture” OR “pharmacopuncture” OR “transcutaneous electrical acupoint”) AND (“randomized controlled trial” OR “randomized clinical trial”).

The search strategy for PubMed is shown in Supplementary Material Table S2, and was modified according to the characteristics of each database. The search terms were translated into Chinese and Korean for study identification in the Chinese and Korean databases.

### 2.3. Eligibility Criteria for Study Selection

#### 2.3.1. Types of Studies

We included all randomized controlled trials (RCTs) that evaluated the effects of acupuncture treatment on emotional problems in women with infertility. We excluded other studies, including non-RCTs, case series, case reports, crossover studies, letters, and laboratory studies.

#### 2.3.2. Participants

Women diagnosed with infertility and emotional problems were included in the study. Emotional problems were defined as anxiety, depression, low self-efficacy, distress, fear, panic, and nervousness. There were no restrictions regarding age, race, nationality, education, or economic status.

#### 2.3.3. Types of Interventions

Acupuncture, acupressure, electroacupuncture, auricular acupuncture, scalp acupuncture, hand acupuncture, pharmacopuncture, and transcutaneous electrical acupoints were included. 

#### 2.3.4. Types of Comparisons

We compared acupuncture with no treatment, a placebo/sham treatment, and conventional treatments. We also included RCTs that compared combination treatment (acupuncture plus conventional treatment) with conventional treatment alone, when the conventional treatment applied to both the groups was identical.

#### 2.3.5. Types of Outcome Measures

##### Primary Outcomes

(1) Emotion-related assessment scales (e.g., State-Trait Anxiety Inventory (STAI), Self-rating Anxiety Scale (SAS), Amsterdam Preoperative Anxiety and Information Scale (APAIS), Hamilton Anxiety-rating Scale (HAS), Self-rating Depression Scale (SDS), Hamilton Depression Rating Scale (HAM-D), Infertility Self-Efficacy scale (ISE), and Fertility Problem Inventory (FPI))

##### Secondary Outcomes

(1) Total effectiveness rate for emotional problems
(2) Quality of life
(3) Clinical pregnancy rate
(4) Adverse events

### 2.4. Data Collection and Analysis

#### 2.4.1. Selection of Studies

Two authors independently reviewed and screened the titles and abstracts of the included studies using the predetermined eligibility criteria to identify eligible studies. Disagreements were resolved through discussions with a third author.

#### 2.4.2. Data Extraction

Two independent reviewers extracted data on the authors’ information, participants, types of emotional problems, randomization, interventions (e.g., acupuncture type, acupuncture point, needle type, insertion depth, insertion angle, needle retention time, treatment period, and treatment frequency), outcomes, and number of treatment-related adverse events. Details regarding the acupuncture treatment and control interventions were extracted based on the revised Standards for Reporting Interventions in Clinical Trials of Acupuncture [31]. Disagreements regarding the extraction were resolved through discussion with a third author.

#### 2.4.3. Assessment of Risk of Bias

Two authors independently evaluated the risk of bias using the Cochrane risk-of-bias assessment tool [32]. The following domains were assessed: random sequence generation, allocation concealment, blinding of participants, blinding of outcome assessors, incomplete outcome data, selective outcome reporting, and other sources of bias. The risk of bias was rated as low, high, or unclear. Disagreements were resolved by consensus with a third reviewer.

#### 2.4.4. Data Synthesis

RevMan Version 5.4 software (The Cochrane Collaboration, 2020) was used to combine the relative risks for dichotomous data and standardized mean differences for continuous data, with 95% confidence intervals. A random-effects model was used to combine the data into relative risks (RRs) or standardized mean differences (SMDs). When a meta-analysis could not be performed, the results of the studies were summarized.

## 3. Results

### 3.1. Study Selection

**Figure 1:**
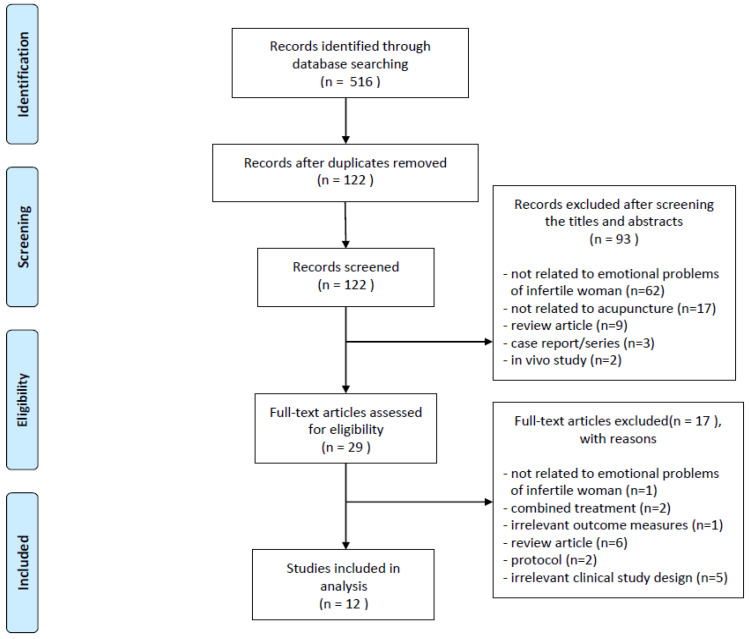
PRISMA flowchart of the study selection process.

After searching the 11 databases, 516 studies were retrieved: 66 from PubMed, 72 from EMBASE, 45 from the Cochrane Library, 120 from CNKI, and 213 from the Wanfang Database. After excluding duplicate studies, the title and abstract of the remaining 122 studies were screened, and 29 studies remained after the initial screening. Subsequently, the full texts of the 29 studies were reviewed, and 17 studies that did not meet the eligibility criteria were excluded: one that is not related to emotional problems of infertile women, two that did not use acupuncture as an intervention method, six review articles, two protocol articles, and five that were not RCTs. Finally, 12 studies were included. The study selection process based on the PRISMA flow diagram is illustrated in Figure 1.

### 3.2. Main Characteristics of the Included Studies

The 12 selected RCTs [33,34,35,36,37,38,39,40,41,42,43,44] were conducted between 2009 and 2020. Six studies [35,37,38,39,41,43] were conducted in China, whereas the others were conducted in the United States [33], Australia [34], Brazil [36], Iran [40], Australia and New Zealand [42], and Turkey [44]. Eight studies [33,34,36,37,39,40,42,44] were written in English, and four studies [35,38,41,43] were written in Chinese.

A total of 1930 women with infertility experiencing emotional problems were included in the 12 RCTs. The experimental group included 866 participants, and the control groups included 1064 participants. None of the 12 studies reported statistically significant differences in general characteristics between the experimental and control groups. 

Regarding the emotional symptoms of women with infertility assessed in each study, four studies [34,35,38,43] involved two or more emotional symptoms, and eight studies [33,36,37,39,40,41,42,44] involved a single symptom. Anxiety was the most commonly investigated emotional symptom and was examined in 10 studies [33,34,35,36,37,38,39,42,43,44], followed by depression in four studies [35,38,41,43], low self-efficacy in two studies [34,40], and infertility-related stress in one study [34]. Table 1 summarizes the details of the included studies.

### 3.3. Interventions

The intervention group was further divided into two subgroups as follows: acupuncture treatment and combined treatment (acupuncture plus conventional treatment). Eleven studies [33,34,35,36,37,38,39,40,42,43,44] used only acupuncture treatment, and one study [41] used a combined treatment (acupuncture treatment plus fluoxetine). 

In the experimental group, manual acupuncture was the most commonly used acupuncture treatment, and it was investigated in eight studies [33,34,35,36,41,42,43,44], followed by transcutaneous electrical acupoint stimulation (TEAS) [38,39] and acupressure [37,40] in two studies, respectively. Acupuncture treatment was performed during the in vitro fertilization–embryo transfer (IVF-ET) process in 10 studies [33,35,36,37,38,39,40,42,43,44]. 

The most frequently used acupuncture point was PC6, which was used nine times; followed by ST36 and SP6 eight times; HT7, GV20, and LR3 seven times; and SP8 and CV4 six times. The acupuncture retention time varied from 3 to 45 min. The most common retention time was 30 min in five studies, followed by 25 min in three studies. The details of the acupuncture interventions are summarized in Table 2.

### 3.4. Control Intervention

The control interventions were classified into three types: no treatment, sham acupuncture treatment, and conventional treatment. Nine studies [33,34,35,37,38,39,40,43,44] used no treatment as a control intervention, five studies [36,37,38,40,42] used sham acupuncture treatment, and one study [41] used conventional treatment (fluoxetine). Three studies [37,38,40] included two control groups: no treatment and sham acupuncture treatment groups. Table 3 shows the characteristics of the sham acupuncture treatments in the control group.

### 3.5. Outcomes

#### 3.5.1. Anxiety-Related Assessment Scales

Among the 12 studies, a total of 10 studies [33,34,35,36,37,38,39,42,43,44] examined anxiety in women with infertility, and the STAI, SAS, APAIS, and HAS were used as anxiety assessment scales.

##### State–Trait Anxiety Inventory (STAI)

**Figure 2:**
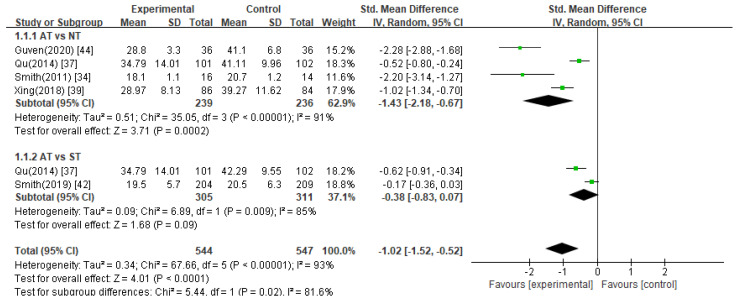
Meta-analysis of STAI scores. AT, acupuncture treatment; NT, no treatment; ST, sham treatment.

In six studies [33,34,37,39,42,44], the STAI was used to evaluate changes in anxiety symptoms before and after treatment. Further, two [37,39] of the six studies comprised two control groups: no treatment and sham treatment. Overall, the meta-analysis of these studies revealed that the STAI score was significantly reduced in the acupuncture treatment group compared to that in the control group (Figure 2, SMD −1.02, 95% CI −1.52 to −0.52).

1. Acupuncture treatment versus no treatment

Acupuncture treatment was compared with no treatment in five studies [33,34,37,39,44], and meta-analysis of the four studies [34,37,39,44] that provided sufficient data for statistical analysis showed that acupuncture significantly reduced STAI scores compared to no treatment (Figure 2, SMD −1.43, 95% CI −2.18 to −0.67). Although one study [33] was not included in the meta-analysis due to insufficient data, it was reported that the acupuncture group achieved a statistically significant effect compared to the no treatment group (*p* = 0.015).

2. Acupuncture treatment versus sham acupuncture treatment

A meta-analysis of two studies [37,42] comparing acupuncture with sham acupuncture showed no significant difference between the acupuncture and sham acupuncture treatment groups in reducing STAI scores in women with infertility (Figure 2, SMD −0.38, 95% CI −0.83 to 0.07).

##### Self-Rating Anxiety Scale (SAS)

**Figure 3:**
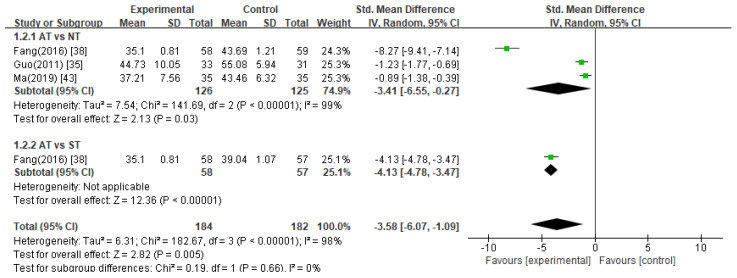
Meta-analysis of SAS scores. AT, acupuncture treatment; NT, no treatment; ST, sham treatment.

Three studies [35,38,43] used the SAS as the assessment scale for anxiety symptoms, and one study [38] included two control groups: no treatment and sham treatment. Overall, the meta-analyses of these studies revealed that patients in the acupuncture group demonstrated significantly reduced SAS scores compared to the control groups (Figure 3, SMD −3.58, 95% CI −6.07 to −1.09).

1. Acupuncture treatment versus no treatment

According to the meta-analysis results of three studies [35,38,43] comparing acupuncture treatment with no treatment, anxiety in women with infertility measured using the SAS significantly decreased in the acupuncture group compared to that of the no treatment group (Figure 3, SMD −3.41, 95% CI −6.55 to −0.27).

2. Acupuncture treatment versus sham acupuncture treatment

One study [38] reported that acupuncture treatment significantly lowered SAS scores compared with sham acupuncture treatment (*p* < 0.05).

##### Amsterdam Preoperative Anxiety and Information Scale (APAIS)

**Figure 4:**
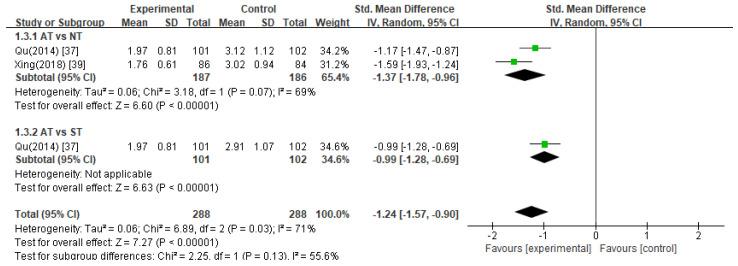
Meta-analysis of APAIS scores (anesthesia-related anxiety). AT, acupuncture treatment; NT, no treatment; ST, sham treatment.

**Figure 5:**
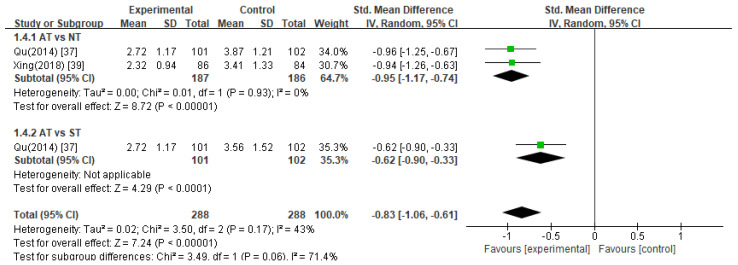
Meta-analysis of APAIS scores (surgery-related anxiety). AT, acupuncture treatment; NT, no treatment; ST, sham treatment.

**Figure 6:**
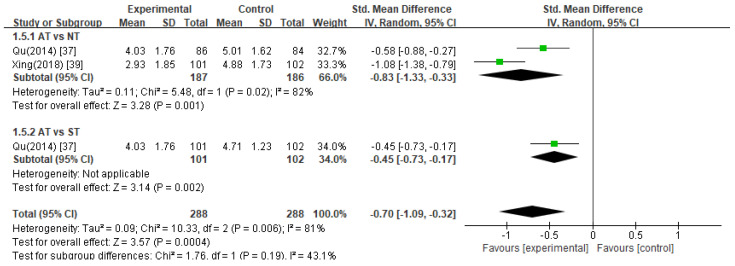
Meta-analysis of APAIS scores (need for information). AT, acupuncture treatment; NT, no treatment; ST, sham treatment.

The pooled data from two studies [37,39] revealed that acupuncture treatment significantly decreased APAIS scores compared to the control treatments in anesthesia-related anxiety (Figure 4, SMD −1.24, 95% CI −1.57 to −0.90), surgery-related anxiety (Figure 5, SMD −0.83, 95% CI −1.06 to −0.61), and the need for information (Figure 6, SMD −0.70, 95% CI −1.09 to −0.32).

1. Acupuncture treatment versus no treatment

In two studies [37,39], acupuncture treatment was compared with no treatment, and a meta-analysis of these studies showed that acupuncture treatment significantly decreased APAIS scores in the areas of anesthesia-related anxiety (Figure 4, SMD −1.37, 95% CI −1.78 to −0.96), surgery-related anxiety (Figure 5, SMD −0.95, 95% CI −1.17 to −0.74), and the need for information compared to the no treatment group (Figure 6, SMD −0.83, 95% CI −1.33 to −0.33).

2. Acupuncture treatment versus sham acupuncture treatment

Compared with sham acupuncture treatment, one study [37] reported that acupuncture treatment significantly reduced APAIS scores (*p* < 0.05).

##### Hamilton Anxiety-Rating Scale (HAS)

1. Acupuncture treatment versus sham acupuncture treatment

One study [36] compared acupuncture treatment with sham acupuncture treatment and reported that acupuncture treatment significantly improved HAS scores compared to sham acupuncture treatment (*p* = 0.0008).

#### 3.5.2. Depression-Related Assessment Scales

Of the twelve studies, four [35,38,41,43] investigated depression in women with infertility, and the SDS and HAM-D were used to evaluate depression.

##### Self-Rating Depression Scale (SDS)

**Figure 7:**
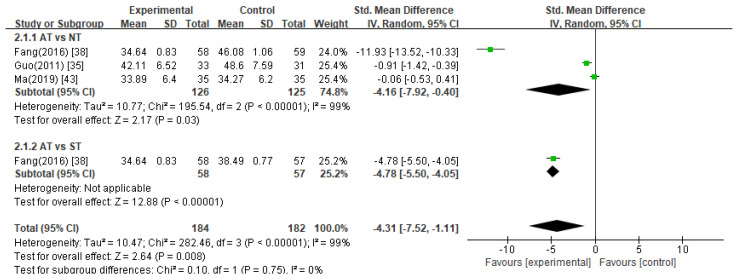
Meta-analysis of SDS scores. AT, acupuncture treatment; NT, no treatment; ST, sham treatment.

Three studies [35,38,43] used the SDS as the assessment scale for evaluating depression, and in one study [38], there were two control groups: no treatment and sham treatment. Overall, the meta-analyses of these studies revealed that acupuncture was significantly more effective in reducing SDS scores than the control treatments (Figure 7, SMD −4.31, 95% CI −7.52 to −1.11).

1. Acupuncture treatment versus no treatment

The pooled data from three studies [35,38,43] showed that the acupuncture treatment group showed a significant decrease in the SDS scores compared to the no treatment group (Figure 7, SMD −4.16, 95% CI −7.92 to −0.40).

2. Acupuncture treatment versus sham acupuncture treatment

One study [38] compared acupuncture treatment with sham acupuncture treatment and reported that acupuncture treatment significantly improved the SDS scores compared with sham acupuncture treatment (*p* < 0.05).

##### Hamilton Depression Rating Scale (HAM-D)

1. Acupuncture plus conventional treatment versus conventional treatment

One study [41] compared acupuncture plus fluoxetine to fluoxetine alone using depression-related assessment scales and reported that acupuncture plus fluoxetine treatment resulted in a statistically significant improvement in the HAM-D scores compared with fluoxetine treatment alone (*p* < 0.05).

#### 3.5.3. Low Self-Efficacy-Related Assessment Scales

Among the twelve studies, two [34,40] evaluated the self-efficacy of women with infertility, and the ISE was used as the evaluation scale. 

##### Infertility Self-Efficacy Scale

**Figure 8:**
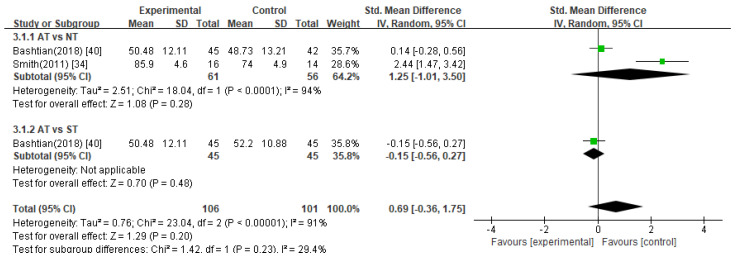
Meta-analysis of ISE scores. AT, acupuncture treatment; NT, no treatment; ST, sham treatment.

Two studies [34,40] evaluated self-efficacy using the ISE, and one study included two control groups: no treatment and sham treatment. In the meta-analysis of these studies, there was no statistically significant difference in ISE between the acupuncture group and the control groups (Figure 8, SMD 0.69, 95% CI –0.36 to 1.75).

1. Acupuncture treatment versus no treatment

The pooled data from two studies [34,40] indicated that there was no significant difference in ISE scores between the acupuncture treatment group and no treatment group (Figure 8, SMD 1.25, 95% CI −1.01 to 3.50).

2. Acupuncture treatment versus sham acupuncture treatment

One study [40] reported no significant difference in ISE scores between the acupuncture and sham treatment groups (*p* > 0.05). 

#### 3.5.4. Infertility-Related Stress Assessment Scales

Of the twelve studies, one [34] investigated infertility-related stress in women with infertility, using the FPI as the evaluation scale.

##### Fertility Problem Inventory (FPI)

1. Acupuncture treatment versus no treatment

One study [34] assessed infertility-related stress using the FPI and reported a statistically significant effect of acupuncture treatment on the ‘relationship concerns’ domain compared to the no treatment group (*p* < 0.05).

#### 3.5.5. Total Effectiveness Rate

##### Acupuncture Plus Conventional Treatment Versus Conventional Treatment

In one study [41], the total effectiveness rate (TER) was evaluated by comparing the HAM-D scores before and after treatment. Acupuncture plus fluoxetine showed a significantly better effect on the TER than fluoxetine alone (*p* = 0.007).

#### 3.5.6. Quality of Life

##### Acupuncture Treatment Versus Sham Acupuncture Treatment

One study [42] assessed quality of life using the 36-Item Short Form Survey (SF-36), and reported that there was no significant difference between the acupuncture and sham treatment groups (*p* > 0.05).

#### 3.5.7. Clinical Pregnancy Rate

**Figure 9:**
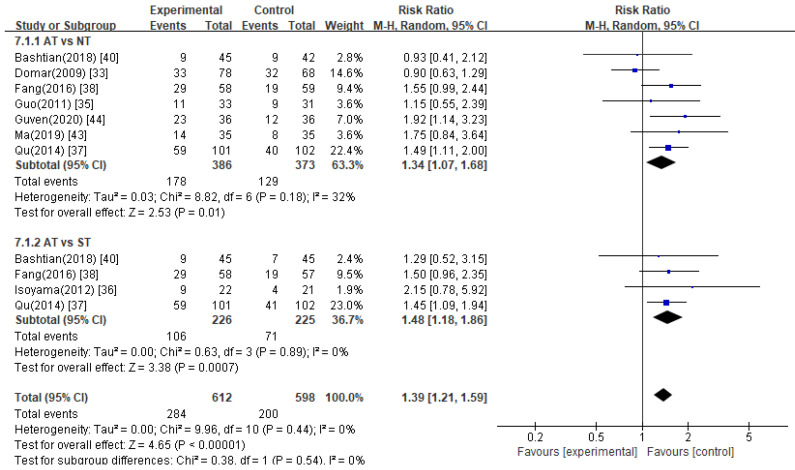
Meta-analysis of clinical pregnancy rate. AT, acupuncture treatment; NT, no treatment; ST, sham treatment.

Eight [33,35,36,37,38,40,43,44] studies compared the clinical pregnancy rate between the acupuncture treatment and control groups, and three [37,38,40] of the eight had two control groups: no treatment and sham treatment. A meta-analysis indicated that the acupuncture treatment group had a statistically higher pregnancy rate than the control groups (Figure 9, RR 1.39, 95% CI 1.21 to 1.59).

##### Acupuncture Treatment Versus No Treatment

Pooled data from seven studies [33,35,37,38,40,43,44] showed that acupuncture treatment had a significant effect on the clinical pregnancy rate compared to no treatment (Figure 9, RR 1.34, 95% CI 1.07 to 1.68). 

##### Acupuncture Treatment Versus Sham Acupuncture Treatment

Pooled data from four studies [33,34,35,37] showed that acupuncture treatment had a significant effect on the clinical pregnancy rate compared to sham treatment (Figure 9, RR 1.48, 95% CI 1.18 to 1.86). 

#### 3.5.8. Adverse Events

Adverse events were mentioned in three studies [37,39,44], but no adverse events were reported in the acupuncture treatment groups.

### 3.6. Assessment for ROB

**Figure 10:**
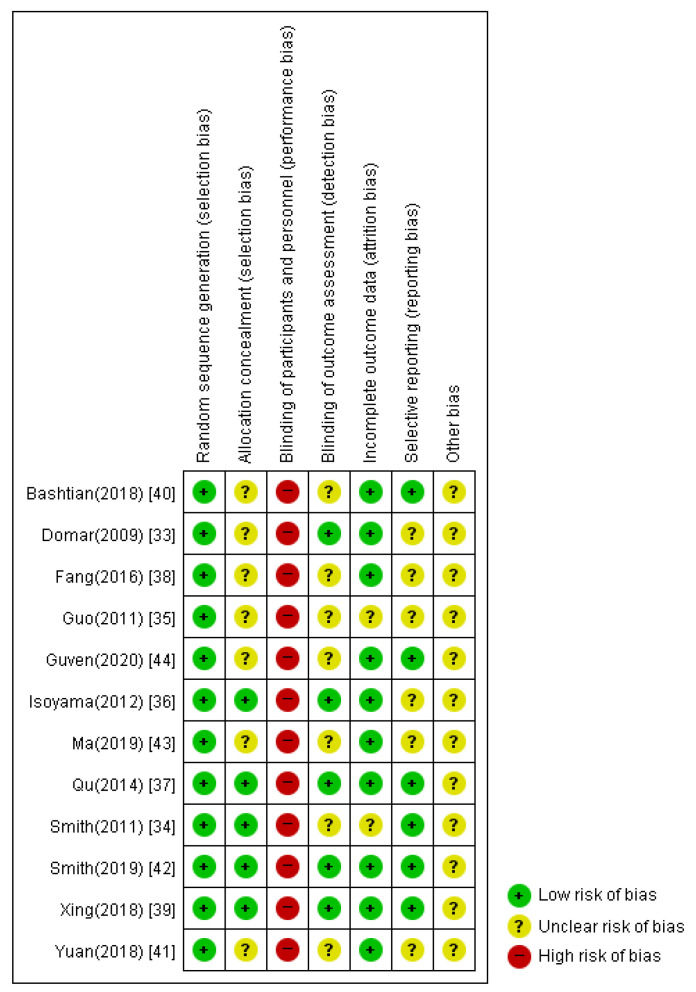
Risk of bias summary.

**Figure 11:**
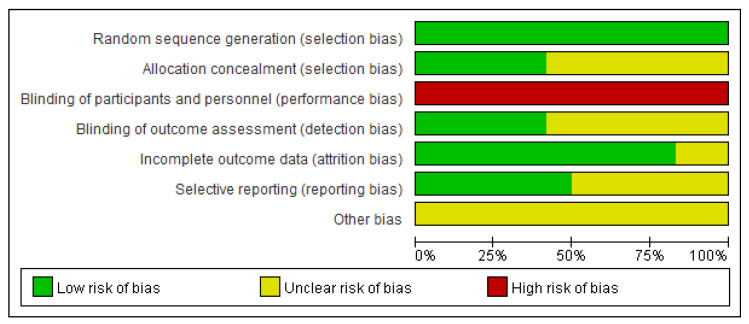
Risk of bias graph.

The risk of bias in the included studies is shown in Figure 10 and Figure 11. Regarding the randomization procedure, 11 studies [33,34,35,36,37,38,39,40,41,42,43] reported an appropriate randomization procedure using a computer random number generator or random number table and were evaluated as low-risk. One study [44] was evaluated as having unclear risk because there was no mention of random sequence generation. 

In five [34,36,37,39,42] of the twelve studies, the allocation order was concealed by an appropriate method; therefore, the risk of bias was evaluated as low. In the other seven studies [33,35,38,40,41,43,44], the risk of bias was unclear because there was no mention of allocation concealment.

Owing to the nature of the intervention, all 12 studies were evaluated as high-risk regarding the blinding of participants and researchers. 

Five [33,36,37,39,42] of the twelve studies reported that the outcome assessment was performed by an independent outcome assessor who remained blind, while the other seven studies [34,35,38,40,41,43,44] did not mention blinding of the outcome assessor. 

Ten studies [33,36,37,38,39,40,41,42,43,44] were evaluated as having low risk. There were no missing data in five studies [33,36,37,41,43], and in four studies [38,39,40,44], missing data occurred similarly in both the experimental and control groups for a similar reason. In one study, an intention-to-treat analysis was performed [42] to minimize attrition bias. The remaining two studies [34,35] were evaluated as having unclear risk.

Six [34,37,39,40,42,44] of the twelve studies were rated as having a low risk of bias in the selective reporting domain because the studies were conducted according to existing protocols. The remaining six studies [33,35,36,38,41,43] did not provide information about the published or registered study protocols.

All 12 studies were evaluated as having unclear risk for other bias items.

## 4. Discussion

### 4.1. Main Findings

This systematic review examined the effectiveness and safety of acupuncture treatment in treating emotional problems in women with infertility. The meta-analysis indicated that acupuncture treatment was more effective in improving the anxiety-related assessment scores of the STAI, SAS, and APAIS and the depression-related assessment SDS scores than the control interventions. However, the meta-analysis showed no statistically significant difference in the ISE scores between the acupuncture treatment and control groups. Although the HAS, HAM-D, and FPI were used in only one study and a statistical analysis could not be performed, individual studies reported that acupuncture treatment had a statistically significant effect on HAS and FPI scores compared to controls and that acupuncture plus fluoxetine was more effective in improving HAM-D scores than fluoxetine alone.

In all the included studies, the three most commonly used acupuncture points for treating emotional problems in women with infertility were PC6, ST36, and SP6. PC6 has been used clinically to treat psychiatric and psychosomatic disorders and is known to exhibit sedative effects against various stresses [45]. ST36 is an acupoint that is widely used to treat gastrointestinal and psychiatric disorders, and recent studies have reported that ST36 exhibits significant anxiolytic effects [46]. SP6 is one of the most common points used in treating psychological dysfunctions, and recent studies have reported the anti-anxiety and antidepressant-like effects of SP6 [47]. 

Regarding safety, no adverse events were reported in the acupuncture treatment groups. Because only three RCTs reported adverse events, this finding should be interpreted with caution. 

A meta-analysis of eight studies reporting clinical pregnancy rates indicated that the acupuncture group achieved a significantly higher pregnancy rate than the control group. These results indicate that acupuncture treatment can be considered safe for women with infertility because it does not negatively affect infertility compared to control interventions but improves pregnancy rates. The limited results of this study cannot clearly determine whether improving emotional symptoms in women with infertility increases the clinical pregnancy rate. Future clinical studies are required to clarify the relationship between the two.

After assessing the quality of the included studies, the risk of bias was generally unclear or low, and because of the nature of the intervention method, the risk of bias was particularly high in performance-bias-related items.

### 4.2. Strength and Limitation

A limitation of this study is that the number of included studies was small, and owing to the heterogeneity of the control interventions and outcome measures, the number of studies included in the statistical pooling for each outcome measure was small. Expert group meetings are required to standardize the assessment scales used in clinical studies based on individual emotional symptoms. Furthermore, as the types of acupuncture treatments, acupuncture points, and regimens of intervention varied across studies, there are limitations in suggesting which specific type of acupuncture or regimens is most effective. In addition, most of the included studies focused on emotional problems in women with infertility during IVF-ET procedures, whereas few focused on negative emotional problems in women with infertility who did not undergo IVF-ET procedures. Additionally, the number of studies included in the meta-analysis was insufficient to generate funnel plots to evaluate the publication bias.

This study is meaningful because it evaluated the effectiveness and safety of acupuncture for emotional symptoms in women with infertility and provides a clinical basis for acupuncture treatment in women with infertility with emotional symptoms. However, the effect of acupuncture on emotional problems in women with infertility observed in this study needs to be confirmed by high-quality clinical studies.

## 5. Conclusions

Our review and meta-analysis suggest that acupuncture may be an effective and safe treatment for emotional problems, particularly anxiety and depression, in women with infertility. However, the evidence is insufficient to confirm the efficacy and safety of acupuncture treatment because of the low methodological quality, heterogeneity of interventions, and small number of included studies. Therefore, well-designed, high-quality RCTs are required to confirm our findings.

## Figures and Tables

**Table 1:** Characteristics of the included studies.

First Author (Year)	Country	Sample Size (EG:CG)	Mean Age (±SD)	Mean Duration of Infertility (±SD, yr)	Emotional Problem(s)	Interventions		Outcome Measurement	Main Results	AEs
						EG	CG			
Domar (2009) [33]	U.S.A.	146 (78:68)	EG: 36.1 CG: 36.1	NR	anxiety	Acupuncture	No treatment	1. STAI 2. CPR	1. positive ^a^ 2. NS	NR
Smith (2011) [34]	Australia	32 (16:16)	EG: 35.1 ± 4.2 CG: 34.1 ± 5.2	EG: 4.50 ± 3.5 CG: 3.57 ± 2.4	anxiety, self-efficacy, infertility-related stress	Acupuncture	No treatment	1. STAI 2. FPI (1) Social concern (2) Sexual concern (3) Relationship concern (4) Rejection of childfree lifestyle (5) Need for parenthood 3. ISE	1. NS 2. (1) NS (2) NS (3) positive ^a^ (4) NS (5) NS 3. NS	NR
Guo (2011) [35]	China	64 (33:31)	EG: 31.49 ± 2.96 CG: 30.93 ± 3.39	EG: 4.02 ± 2.13 CG: 3.86 ± 1.74	anxiety, depression	Acupuncture	No treatment	1. SAS 2. SDS 3. CPR	1. positive ^b^ 2. positive ^b^ 3. NS	NR
Isoyama (2012) [36]	Brazil	43 (22:21)	EG: 34.1 ± 4.6 CG: 34.3 ± 4.6	EG: 3.4 ± 1.5 CG: 4.7 ± 2.8	anxiety	Acupuncture	Sham Acupuncture	1. HAS 2. CPR	1. positive ^c^ 2. positive ^b^ 3. NS	NR
Qu (2014) [37]	China	305 (101:102:102)	EG: 31.65 ± 4.30 CG1: 30.87 ± 4.12 CG2: 30.95 ± 4.78	EG: 5.19 ± 3.39 CG1: 4.16 ± 3.21 CG2: 4.97 ± 4.17	anxiety	Acupressure	CG1: Sham Acupressure CG2: No treatment	1. STAI 2. APAIS 3. CPR	1. positive ^a(d,e)^ 2. positive ^a(d,e)^ 3. positive ^b(d,e)^	None
Fang (2016) [38]	China	180 (60:60:60)	EG: 29.69 ± 0.47 CG1: 29.53 ± 0.48 CG2: 30.39 ± 0.55	NR	anxiety, depression	TEAS	CG1: Sham TEAS CG2: No treatment	1. SAS 2. SDS 3. CPR	1. positive ^a(d,e)^ 2. positive ^a(d,e)^ 3. positive ^a(d,e)^	NR
Xing (2018) [39]	China	180 (90:90)	EG: 31.99 ± 4.56 CG: 31.24 ± 4.91	EG: 5.55 ± 4.42 CG: 5.32 ± 3.91	anxiety	TEAS	No Treatment	1. STAI 2. APAIS	1. positive ^a^ 2. positive ^a^	None
Bashtian (2018) [40]	Iran	132 (45:45:42)	EG: 30.20 ± 2.56 CG: 31.08 ± 2.87	3.89 ± 3.80	self-efficacy	Acupressure	CG1: Sham Acupressure CG2: No treatment	1. ISE 2. CPR	1. NS ^(d,e)^ 2. NS ^(d,e)^	NR
Yuan (2018) [41]	China	98 (49:49)	EG: 35.7 ± 4.3 CG: 35.7 ± 4.2	EG: 15.87 ± 2.16(mo) CG: 16.33 ± 1.81(mo)	depression	Acupuncture + CG Treatment	Fluoxetine 20 mg	1. HAM-D 2. TER	1. positive ^a^ 2. positive ^b^	NR
Smith (2019) [42]	Australia, New Zealand	608 (301:307)	EG: 30.04 ± 2.98 CG: 30.55 ± 3.71	NR	anxiety	Acupuncture	Sham Acupuncture	1. STAI 2. SF-36	1. positive ^a^ 2. NS	NR
Ma (2019) [43]	China	70 (35:35)	EG 30.70 ± 5.14 CG 29.98 ± 4.95	EG: 4.4 ± 1.8 CG: 4.9 ± 1.5	anxiety, depression	Acupuncture	No treatment	1. SAS 2. SDS 3. CPR	1. positive ^a^ 2. NS 3. positive ^a^	NR
Guven (2020) [44]	Turkey	72 (36:36)	EG: 30.3 ± 3.4 CG: 31.5 ± 4	NR	anxiety	Acupuncture	No treatment	1. STAI 2. CPR	1. positive ^c^ 2. positive ^b^	None

^a^ *p* < 0.05; ^b^ *p* < 0.01; ^c^ *p* < 0.001; ^d^ EG compared with CG1; ^e^ EG compared with CG2. EG, experimental group; CG, control group; SD, standard deviation; yr, year; mo, month; AEs, adverse events; NR, not recorded; NS, no significant difference; TEAS, transcutaneous electrical acupoint stimulation; STAI, State–Trait Anxiety Inventory; CPR, clinical pregnancy rate; FPI, Fertility Problem Inventory; ISE, Infertility Self-Efficacy scale; SAS, Self-rating Anxiety Scale; SDS, Self-rating Depression Scale; HAS, Hamilton Anxiety-rating Scale; APAIS, Amsterdam Preoperative Anxiety and Information Scale; HAM-D, Hamilton Depression Rating Scale; TER, total effectiveness rate; SF-36, The 36-Item Short Form Survey.

**Table 2:** Characteristics of acupuncture interventions in the included studies.

First Author (Year)	TYPE of Acupuncture	Regimen	Number of Needle Insertions	Acupuncture Points	Depth of Insertion	Response Sought	Needle Stimulation (Frequency)	Needle Retention Time	Types of Needle (Diameter, Length)	Co-Interventions
Domar (2009) [33]	Acupuncture	2 sessions (① before ET, ② after ET)	12–13	① PC6, SP8, LR3, GV20, ST29, Ear points (TF4, CO18, TF2, AT3) (uni) ② ST36, SP6, SP10, LI4, Ear points (TF4, CO18, TF2, AT3) (uni)	10–20 mm	de qi	manual	25 min	0.25 mm, 25 mm/ 0.2 mm, 13 mm (ear points)	none
Smith (2011) [34]	Acupuncture	6 sessions (8 weeks)	3–11	usually PC5, PC6, HT5, HT7 (points selected in response to emotional complaints)	NR	de qi	manual	45 min	0.2 mm, 30 mm	none
Guo (2011) [35]	Acupuncture	NR (from the day of downregulation until the HCG injection)	7	CV3, LR3, EX-CA1, SP6	CV3(1–1.5 cun), LR3(1–1.5 cun), EX-CA1 (1.5–2 cun), SP6(0.5–1.0 cun)	de qi	manual	NR	NR, 40 mm	none
Isoyama (2012) [36]	Acupuncture	4–6 sessions (once a week, during the process from ovulation induction to the result of β-hcG)	7	HT7, PC6, CV17, GV20, EX-HN3	NR	de qi	manual	25 min	0.25 mm, 40 mm	none
Qu (2014) [37]	Acupressure	24 sessions (4 times/day, 6 days (from 1 day before TVOR to the next day of ET))	6	Ear points (TF4, CO18, TF2)	NR	NR	NR	15 min	NR	none
Fang (2016) [38]	TEAS	NR (from the day of downregulation until the HCG injection)	8	HT7, PC6, ST36, SP6	NR	NR	electrical (2/15 Hz)	30 min	NR	none
Xing (2018) [39]	TEAS	2 sessions (① 24 h before TVOR ② 2 h before ET)	6–8	① SP10, SP8, LR3, ST36 ② EX-CA1, RN4, PC6, CV12	NR	visible muscle contraction response	electrical (2/100 Hz)	30 min	NR	none
Bashtian (2018) [40]	Acupressure	12 sessions (4 sessions/week, until the day before ET)	4	PC6, HT7	NR	feeling of heaviness	NR	3 min	NR	none
Yuan (2018) [41]	Acupuncture	28 sessions (daily, 28 days)	10–14	GV20, EX-HN3, PC6, SP6, CV3, CV4, EX-CA1 (Deficiency of both the heart and spleen: add ST36, HT7/ Liver-qi stagnation: add LR3, LI4)	NR	NR	manual	30 min	NR	CG Treatment (Fluoxetine 20 mg)
Smith (2019) [42]	Acupuncture	3 sessions (① between day 6 and 8 of ovarian stimulation ② 1 h before ET ③ following ET)	8–13	① ST29, CV4, CV6, SP6 SP10 ② ST29, SP8, SP10, LR3, CV4, ear point (TF2), one of three (HT7, PC6 or EX-HN3) ③ GV20, KI3, ST36, SP6, PC6, ear point (TF4)	NR	de qi	manual	25 min	0.35 mm, 70 mm	none
Ma (2019) [43]	Acupuncture	NR (① every other day (from the 2nd day of menstruation until the day of ET) ② 30 min after ET)	12–18	① ST25, CV4, CV6, ST29, ST36, SP8, EX-CA1, SP6, LR3, GV20, EX-HN3 ② ST25, CV4, CV6, ST29, ST36, SP8, KI3	NR	de qi	manual	30 min	0.25 mm, 40 mm	none
Guven (2020) [44]	Acupuncture	3 sessions (① 1 week before ET ② 30 min before ET ③ 30 min after ET)	7–10	① HT7, LI4, GV20, Ear point (TF4) ② CV3, CV4, CV6, GV20, LR3, ST30, SP8 ③ LI4, SP6, SP9, ST36	1–2 cun	NR	none	30 min	0.25 mm, 25 mm	none

NR, not reported; TVOR, transvaginal oocyte retrieval; ET, embryo transfer; HCG, Human Chorionic Gonadotropin; uni, unilateral.

**Table 3:** Characteristics of sham acupuncture interventions in the included studies.

First Author (Year)	Type of Acupuncture	Regimen	Number of Needle Insertions (per Session)	Acupuncture Points	Depth of Insertion	Response Sought	Needle Stimulation (Frequency)	Needle Retention Time	Types of Needle (Diameter, Length)
Isoyama (2012) [36]	Sham Acupuncture	4–6 sessions (once a week, during the process from ovulation induction to the result of β-hcG)	7	located close to but not on the real acupuncture points (distance of approximately 1.5 cm in regions)	2 mm	none	none	25 min	0.25 mm, 40 mm
Qu (2014) [37]	Sham Acupressure	24 sessions (4 times/day, 6 days (from 1 day before TVOR to the next day of ET))	6	Ear points (CO17, CO4, CO7)	NR	NR	NR	15 min	NR
Fang (2016) [38]	Sham TEAS	NR (from the day of downregulation until the HCG injection)	8	HT7, PC6, ST36, SP6	NR	NR	electrical (NR)	30 min	Sham TEAS (frequency which has no therapeutic effect)
Bashtian (2018) [40]	Sham Acupressure	12 sessions (4 sessions/week, until the day before ET)	4	2 cm distance of the main points	NR	NR	NR	3 min	NR
Smith (2019) [42]	Sham Acupuncture	3 sessions (① between day 6 and 8 of ovarian stimulation ② 1 h before ET ③ following ET)	6	sham points at locations away from known acupuncture points and with no known function	NR	NR	NR	25 min	0.35 mm, 70 mm (sham needle (non-insertive): Park Sham Device)

NR, not reported; TVOR, transvaginal oocyte retrieval; ET, embryo transfer; HCG, Human Chorionic Gonadotropin.

## Data Availability

The datasets used and/or analyzed during the current study are available from the corresponding author upon reasonable request.

## Supplementary Materials

The following supporting information can be downloaded at https://www.mdpi.com/article/10.3390/healthcare11202704/s1: Table S1: PRISMA 2020 checklist; Table S2: Search strategy used in PubMed.
