# Supplementary material for: Acupuncture Treatment for Emotional Problems in Women with Infertility: A Systematic Review and Meta-Analysis
Source: Healthcare (Basel). 2023 Oct 10;11(20):2704. doi: 10.3390/healthcare11202704 (PMC10606758; doi:10.3390/healthcare11202704)
Supplement: Supplementary file 1 [file healthcare-11-02704-s001.zip › healthcare-2639421 - Supplementary Table S2.pdf]

Supplementary Table S2. Search strategy used in PubMed.

#1	Infertility [Mesh]
#2	infertility [tiab] OR subfertility [tiab] OR subfertile [tiab] OR oligospermia [tiab] OR azoospermia [tiab] OR obstructive azoospermia[tiab] OR genital disease[tiab]
#3	#1 OR #2
#4	Acupuncture [Mesh] OR Acupuncture Therapy [mesh]
#5	acupuncture [tiab] OR acupressure [tiab] OR electroacupuncture [tiab] OR auricular acupuncture [tiab] OR scalp acupuncture[tiab] OR hand acupuncture[tiab] OR pharmacopuncture [tiab] OR transcutaneous electricalacupoint[tiab]
#6	#4 OR #5
#7	Emotions [Mesh] OR Anxiety [Mesh] OR Depression[Mesh] OR Stress, Psychological [Mesh] OR Pain [Mesh] OR Fear [Mesh] OR Panic [Mesh] OR Self Efficacy [Mesh] OR Resilience, Psychological [Mesh] OR Relaxation [Mesh] OR Adaptation, Psychological [Mesh] OR Mental Disorders [Mesh]
#8	emotion [tiab] OR emotional [tiab] OR mood [tiab] OR feeling [tiab] OR psychological [tiab] OR personality [tiab] OR anxiety [tiab] OR anxious [tiab] OR anxiousness [tiab] OR depression [tiab] OR depressive [tiab] OR stress [tiab] OR distress [tiab] OR distressing [tiab] OR pain [tiab] OR painful [tiab] OR fear [tiab] OR panic [tiab] OR nervousness [tiab] OR self-efficacy [tiab] OR relaxation [tiab] OR adaptation [tiab] OR mental disorder [tiab]
#9	#7 OR #8
#10	Randomized Controlled Trial[Mesh] OR Controlled Clinical Trial [Mesh]
#11	randomized controlled trial [tiab] OR randomized clinical trial [tiab] OR randomized [tiab] OR randomly [tiab] OR trial [tiab]
#12	#10 OR #11
#13	#3 AND #6 AND #9 AND #12
